# Myst2/Kat7 histone acetyltransferase interaction proteomics reveals tumour-suppressor Niam as a novel binding partner in embryonic stem cells

**DOI:** 10.1038/s41598-017-08456-2

**Published:** 2017-08-15

**Authors:** Mercedes Pardo, Lu Yu, Shihpei Shen, Peri Tate, Daniel Bode, Blake L. Letney, Dawn E. Quelle, William Skarnes, Jyoti S. Choudhary

**Affiliations:** 1 Proteomic Mass Spectrometry, Wellcome Trust Sanger Institute, Wellcome Genome Campus, Hinxton, Cambridge CB10 1SA United Kingdom; 2 Stem Cell Engineering, Wellcome Trust Sanger Institute, Wellcome Genome Campus, Hinxton, Cambridge CB10 1SA United Kingdom; 3 Departments of Pharmacology and Pathology, The University of Iowa and Holden Comprehensive Cancer Center, Iowa City, IA 52242 USA; 4 Cold Genesys Inc., Santa Ana, CA USA; 5 Wellcome Trust PhD Program, Cambridge Stem Cell Institute, Cambridge, Cambridgeshire United Kingdom

## Abstract

MYST histone acetyltransferases have crucial functions in transcription, replication and DNA repair and are hence implicated in development and cancer. Here we characterise Myst2/Kat7/Hbo1 protein interactions in mouse embryonic stem cells by affinity purification coupled to mass spectrometry. This study confirms that in embryonic stem cells Myst2 is part of H3 and H4 histone acetylation complexes similar to those described in somatic cells. We identify a novel Myst2-associated protein, the tumour suppressor protein Niam (Nuclear Interactor of ARF and Mdm2). Human NIAM is involved in chromosome segregation, p53 regulation and cell proliferation in somatic cells, but its role in embryonic stem cells is unknown. We describe the first Niam embryonic stem cell interactome, which includes proteins with roles in DNA replication and repair, transcription, splicing and ribosome biogenesis. Many of Myst2 and Niam binding partners are required for correct embryonic development, implicating Myst2 and Niam in the cooperative regulation of this process and suggesting a novel role for Niam in embryonic biology. The data provides a useful resource for exploring Myst2 and Niam essential cellular functions and should contribute to deeper understanding of organism early development and survival as well as cancer. Data are available via ProteomeXchange with identifier PXD005987.

## Introduction

The MYST protein family are catalytic subunits of histone acetyltransferase (HAT) complexes. There are five mammalian MYST proteins, namely Myst1/Mof/Kat8, Myst2/Hbo1/Kat7, Myst3/Moz/Kat6a, Myst4/Morf/Kat6b and Tip60/Kat5. The MYST catalytic domain that defines the family contains a C2HC zinc finger and an acetyl-CoA binding site. Individual MYST proteins additionally display other domains such as chromodomains, PHD and zinc fingers^1^. These enzymes play key roles in transcription regulation, and DNA replication, recombination and repair, and consequently are involved in development and a variety of diseases including cancer^1–5^.

All MYST HATs have essential and separate roles in mammalian development^6^. Mouse Myst2 was recently shown to be required for H3K14 acetylation and normal expression of developmental genes during embryonic development^6, 7^. Mouse Mof has similarly been found to be a key regulator of self-renewal and pluripotency through its role in the core embryonic stem cell (ESC) transcriptional network, by H3K4 acetylation of key regulatory loci^8^.

**Figure 1:**
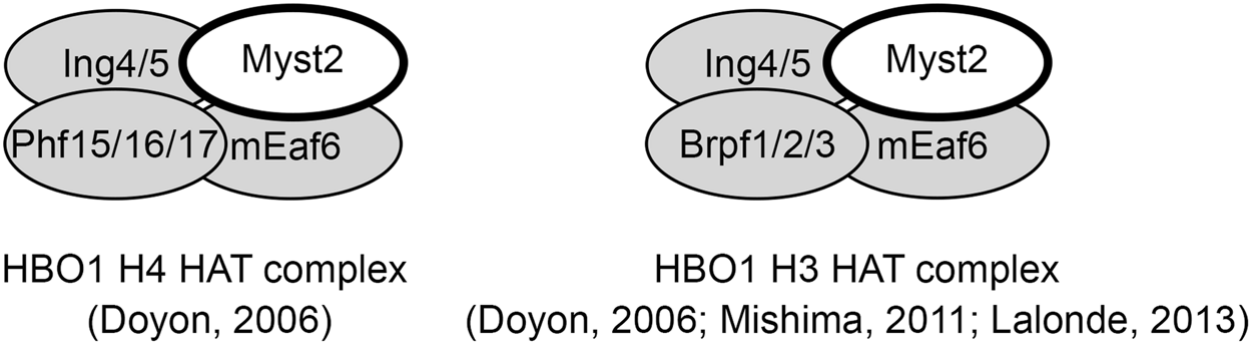
Schematic drawing of H4 and H3 HAT Myst2 complexes. The H4 HAT complex contains Myst2, Eaf6, one of either Ing4 or Ing5, and one of Phf17/Jade1, Phf16/Jade3 or Phf15/Jade2. The H3 HAT complex contains Myst2, Eaf6, one of either Ing4 or Ing5, and one of Brpf1, Brpf2/Brd1 or Brpf3. References describing the complexes are indicated.

MYST HAT complexes are conserved from yeast to human. They are composed of a tetrameric core containing one MYST protein and different subunits with domains that bind chromatin marks^9^. These subunits include members of the ING, BRPF and JADE families and EAF6 (Fig. 1). The MYST2 HAT is a major mediator of both histone H3 (K14, K23) and H4 (K5, K8, K12) acetylation *in vivo* ^1, 6, 7, 10^. The various scaffold subunits of Myst2 HAT complexes control the specificity of histone tail modification: JADE proteins target acetylation onto H4 tails whilst BRPF directs it towards H3^11, 12^. MYST2/HBO1 associates in a combinatorial manner with paralogs JADE1-2-3, the human tumour suppressor proteins ING4 and ING5 and EAF6 in a complex responsible for the majority of histone H4 acetylation in higher eukaryotes^9^. In contrast, paralogs BRPF1-2-3 associate with ING5 and EAF6 to form a MOZ/MORF H3 HAT complex^9, 13^. BRPF2/BRD1 has also been reported to form a MYST2 HAT complex together with ING4 and EAF6 in leukemic cells^7^ and recently BRPF3 was shown to associate with MYST2 to activate its H3 HAT activity and regulate DNA replication^12, 14^. ING, BRPF and JADE proteins contain PHD domains that mediate binding to histones and restrict the substrate specificity of the different MYST HAT complexes^11, 15, 16^.

In human cancer cell lines JADE1 can exist as long (JADE1L) and short (JADE1S) isoforms^9, 17^. JADE1L functions as a scaffold of the MYST2 HAT complex, bringing together MYST2, EAF6 and ING proteins. JADE1S seems to regulate the assembly of the complex by modulating the binding of ING4-5 proteins, suggesting that functionally different forms of the complex containing or not the ING proteins exist^16^. Interestingly, MYST2, JADE1L and JADE1S associate with chromatin during interphase but are excluded from it during mitosis^18^. In human cells BRPF proteins seem to function similarly, bridging the interaction between MYST2 and ING4/5^7, 9, 13^. Mammalian EAF6 protein function has not been elucidated in detail, but yeast Eaf6 is essential for the integrity of yeast NuA4 histone acetyltransferase complex^19^.

Certain roles of MYST2 are independent of histone acetylation and accordingly MYST2 is known to acetylate several substrates other than histones^3^. Acetylation of non-histone substrates can have an effect on their stability, activity, localisation or interaction with other proteins. MYST2 has been shown to associate with and acetylate several pre-replicative complex subunits, namely ORC1, ORC2, MCM2, CDC6 and geminin, and indeed MYST2 has a role not only in replication licensing through the regulation of pre-replicative complex assembly, but possibly also in the activation of origins of replication^12, 14^ and in replication fork movement^20–22^.

In order to gain insight into Myst2 role in embryonic stem cells we created a mouse ESC line where *Myst2* is epitope-tagged at the endogenous locus through targeted gene targeting. We show that in mouse ESCs Myst2 forms HAT complexes with Jade, Brpf, Ing and Meaf6 similar to those described in somatic cells. Furthermore, we identify a novel association between Myst2 and Niam, a poorly understood tumour-suppressor protein linked to the p53 pathway. Expansion of the protein interaction network around Niam in ESCs provides a global picture suggesting previously unobserved roles for this protein.

## Results

### Identification of Myst2 histone acetylation complexes in pluripotent cells

To identify the proteins interacting with Myst2 we generated mouse embryonic stem cells expressing epitope-tagged Myst2 using a gene targeting strategy. The FTAP tag^23, 24^ was introduced at the carboxy terminus of the *Myst2* open reading frame at the endogenous locus, just prior to the stop codon (Supplementary Fig. S1). Correctly targeted clones were identified by long range PCR amplification of genomic DNA using primers external to the homology arms of the vector (Supplementary Fig. S1).

**Figure 2:**
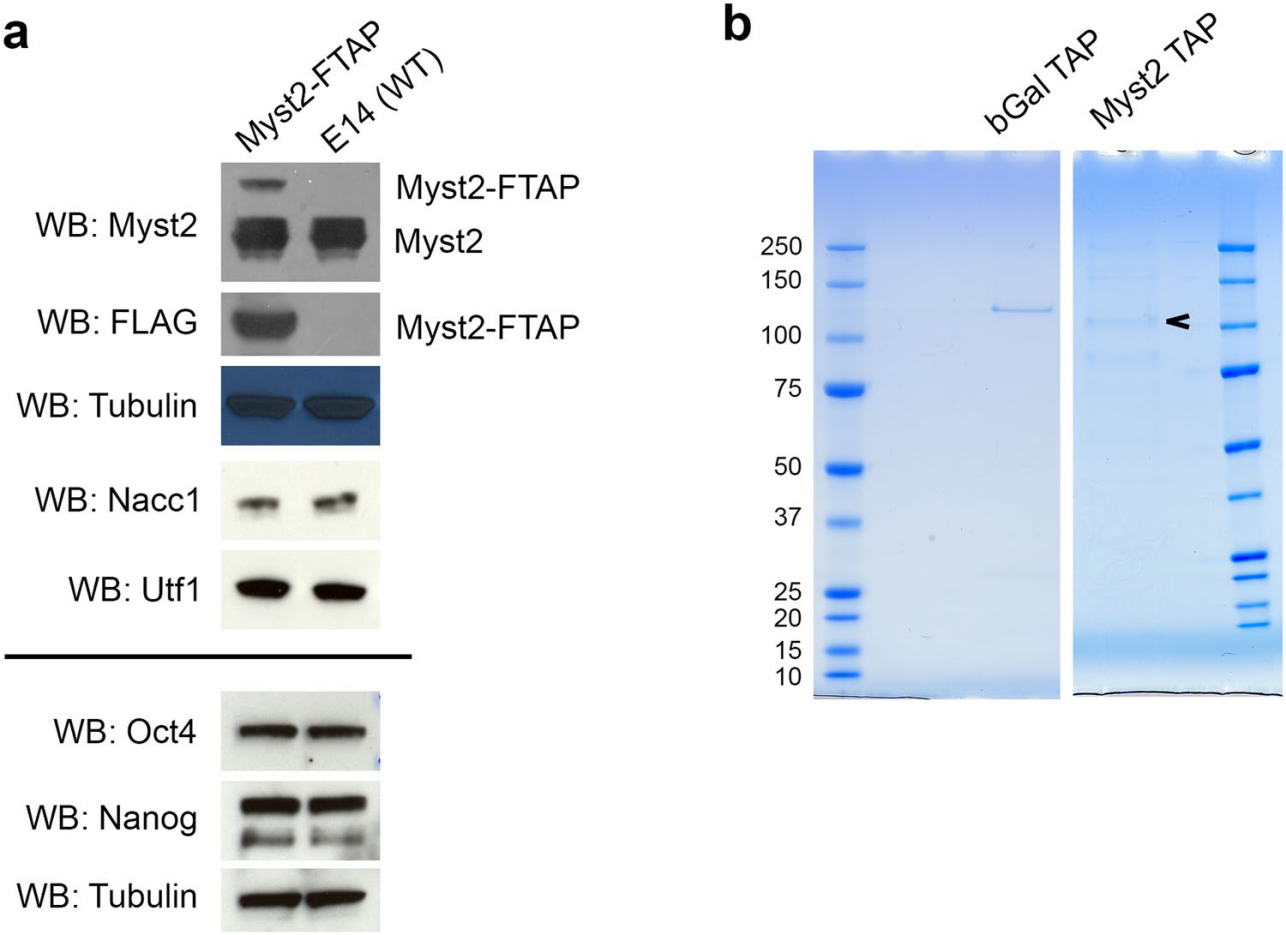
Affinity purification of Myst2 protein complexes from mouse ES cells. (**a**) Whole cell lysates of Myst2-FTAP and wild type E14 cells were probed by Western blotting (WB) with antibodies against Myst2, FLAG, beta-tubulin, Nacc1, Utf1, Oct4 and Nanog. Full-length blots are presented in Supplementary Fig. S3. (**b**) Coomassie-stained gels of proteins co-purifying with Myst2 or control beta-gal in ES cells in tandem affinity purification (TAP). The band corresponding to tagged Myst2 is marked with an arrow. Molecular weight markers are shown (in kDa).

We examined expression of tagged Myst2 by probing whole cell extracts from tagged and untagged cells with anti-FLAG and anti-Myst2 antibodies (Fig. 2a). We detected endogenous Myst2 as a band at the expected molecular weight (75 kDa), with the same intensity in tagged and untagged cells as measured by densitometry analysis of the blot images. In the two tagged clones analysed, an extra anti-FLAG reactive band was detected just below 100 kDa, corresponding to tagged Myst2. The expression of tagged Myst2 was between 20 and 30% that of untagged Myst2, suggesting that the insertion of the tagging cassette has an effect on the regulation of Myst2 protein levels. This however is not a generalised effect, since when tagging other genes using the same cassette and procedure, the tagged clones expressed equivalent amounts of native and tagged protein (P. Tate and M. Pardo, unpublished results). Targeted clones were morphologically indistinguishable from wild type cells (Supplementary Fig. 1c) and expressed markers of ESCs at similar levels as wild type ESCs (Fig. 2a), suggesting that the expression of tagged Myst2 does not interfere with the ESC phenotype.

To identify proteins associated with Myst2 in ESCs we performed three replicate tandem affinity purifications (TAP) of both Myst2-FTAP and a similarly tagged non-relevant protein as control (beta-galactosidase). PAGE separation and staining of the eluted complexes showed several bands specific to Myst2-FTAP purifications (Fig. 2b). Whole lanes were analysed by mass spectrometry to identify the purified proteins. Myst2 was conclusively identified from all Myst2 TAP samples with high sequence coverage, and was absent from control samples. After removal of external contaminants, we obtained a list of 21 proteins identified specifically in all three replicate Myst2 tandem affinity purifications and not present in control experiments (Supplementary Table S1).

Orthologues of all members of the previously described human HBO1 H4 HAT complex were identified from Myst2 TAP samples, namely the ING proteins Ing4 and Ing5, the JADE proteins Phf15, Phf16 and Phf17, and mEaf6 (Supplementary Table S1). Phf17/Jade1 was the most abundant of the JADE proteins, followed by Phf16 and Phf15 in a proportion 6:3:1. The long isoform Jade1l was conclusively identified, with a subset of the peptides also matching the short isoform. This indicates that Myst2-Ing-Jade H4 HAT complexes exist in mouse ESCs.

We also identified two proteins of the BRPF family, the mouse orthologs of BRPF2 and BRPF3, which have also recently been shown to associate with MYST2 in human somatic cells^7, 12, 14^. Brpf3 was by far the most abundant of the BRPF proteins in the set, with Brpf2/Brd1 identified by a smaller number of peptides (Supplementary Table S1). Brpf1 was only identified by peptides shared with Brpf2 and Brpf3, and was therefore not considered in our analysis, in accordance with the Occam’s razor rule, since its presence cannot be conclusively supported by evidence. This indicates that a Brpf3-containing Myst2 H3 HAT complex is the major form in mouse embryonic cells, whilst the Brpf2-containing Myst2 H3 HAT complex is much less abundant. Interestingly, Brpf3 knockdown in somatic cells results in replication defects whilst lack of Brpf2 or Brpf1 does not^12^. Consistent with a role for Myst2 in H3/H4 acetylation, we also specifically detected both histones H3 and H4 amongst interacting partners (Supplementary Table S1). In summary, our data represents the first description of Hbo1/Myst2 HAT complexes in ESCs.

In addition to histone acetylation complexes, Myst2 reproducibly co-purified with the casein kinase II complex subunits (Supplementary Table S1). Casein kinase II plays a global role in chromatin remodelling and cell cycle control^25^ and is involved in the phosphorylation of histone H4 and chromatin remodelling factors^26, 27^.

### Niam is a novel Myst2 interacting protein

We reproducibly detected the Niam protein (historically called Tbrg1 for ‘Transforming growth factor beta regulator 1′ mRNA) amongst Myst2-associated proteins (Supplementary Table S1), suggesting that the interaction with Myst2 is fairly robust since it survives the stringent conditions of tandem affinity purification. NIAM was originally identified as a *n*uclear protein that *i*nteracts with *A*RF and *M*DM2, major regulators of p53^28^. NIAM re-localises ARF, a tumour suppressor and key activator of p53, from the nucleolus to the nucleoplasm, consequently stabilising and activating p53, and so displays anti-proliferative activity. Niam was also recently shown to bind to the MYST protein Tip60 in mouse embryonic fibroblasts^29^.

**Figure 3:**
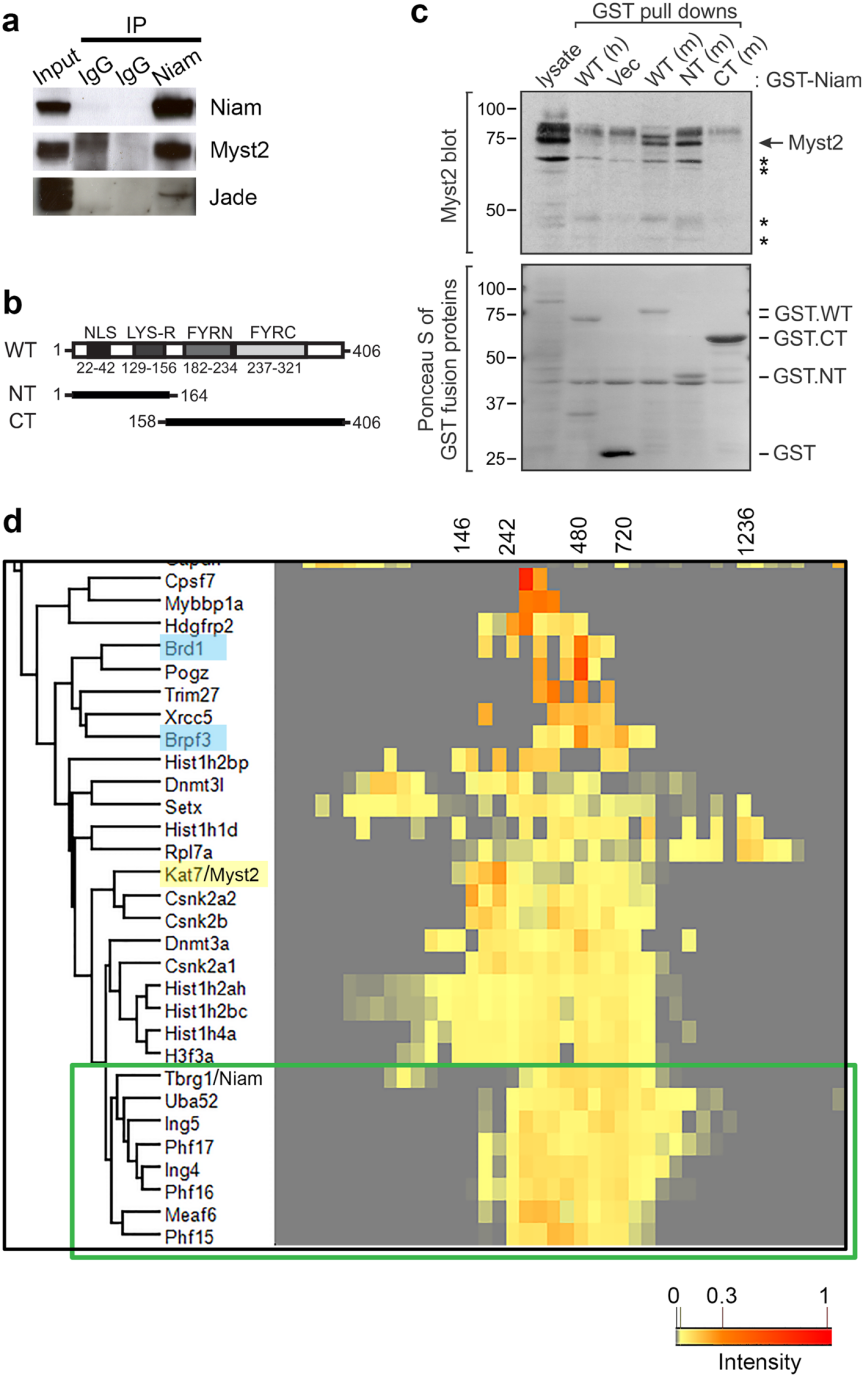
Niam is a novel Myst2-associated protein. (**a**) Validation of the interaction between Myst2 and Niam by reverse co-immunoprecipitation. Endogenous Niam was immunoprecipitated from lysates of wild type ESCs, and the immunoprecipitate was probed with antibodies for the presence of native Myst2. Full-length blots are presented in Supplementary Fig. S4. (**b**) Schematic of wild-type (WT) mouse Niam and deletion mutants NT (residues 1–164) and CT (residues 158–406). The predicted nuclear localization sequence (NLS), lysine-rich region (LYS-R) and ‘FY’ rich regions in the C-terminus are shown. (**c**) Endogenous Myst2 in mESC lysates interacts *in vitro* with GST-tagged WT and NT forms of mouse (m) Niam, but not the CT mutant or GST control. GST-tagged human (h) Niam failed to bind full-length Myst2 (arrow). Asterisks indicate Myst2 antibody-detected bands of lower MW that displayed more selective binding to WT (m and h) and NT Niam. GST protein levels were assessed by Ponceau S staining of the membrane. (**d**) Hierarchical clustering of Myst2-copurifying proteins according to the similarity of their blue native PAGE migration profile. The heatmap represents protein intensities across all the fractions. The green box encloses Myst2 H4 HAT (JADE) complex subunits. Subunits of Myst2 H3 HAT (BRPF) complex are highlighted in light blue. The annotated molecular weights (kDa) were estimated from the migration distances of standards run in parallel.

Given that an interaction between Niam and Myst2 has never been described before, we went on to validate this finding by co-immunoprecipitation of the endogenous untagged proteins. Niam is easily detected in whole cell extracts of mouse embryonic stem cells by western blot using monoclonal antibodies, indicating that it is reasonably well expressed in this cell type (Fig. 3a). Niam was immunoprecipitated from wild type cells using a specific polyclonal antibody and was absent from control rabbit IgG immunoprecipitates (Fig. 3a). Endogenous Myst2 was found to co-immunoprecipitate with Niam. We probed the same blots with antibodies against Phf17, a JADE family protein that is present in Myst2 H3 HAT complexes, and detected a band specific to the Niam IP and not present in the control IP at the expected molecular weight (Fig. 3a). However, based on mass spectrometry results (see below) this band is suspected to correspond to Phf16 and not to Phf17, suggesting that the anti-Phf17 antibody cross-reacts. These results show that Niam indeed associates with Myst2, possibly in a multiprotein complex also containing a JADE family protein.

Two deletion mutants of mouse Niam were used in *in vitro* binding assays to identify domains within Niam that mediate its association with Myst2 (Fig. 3b). The N-terminal (NT) mutant contains residues 1–164 and is responsible for most of Niam’s known functions, including chromatin association, binding to its partners (ARF, MDM2 and Tip60), nuclear localization, p53 activation and growth inhibition^29^. The C-terminal (CT) form of Niam, which represents residues 158–406 and contains the two phenylalanine and tyrosine-rich (FY) domains found in various chromatin binding proteins, lacks the above activities but may contribute to Niam protein stability. Binding assays using GST-tagged Niam proteins (wild-type [WT], NT and CT) mixed with mESC lysates showed that WT and NT forms of mouse Niam, but not the CT mutant or GST control, form complexes with endogenous Myst2 (Fig. 3c). Interestingly, GST-tagged human Niam showed no association with full-length Myst2 but appeared to bind selectively (as did WT and NT mouse Niam) to smaller MW proteins detected by the Myst2 antibody. These assays demonstrate that the functional N-terminal domain of Niam is both required and sufficient for Myst2 binding.

We next tried to establish which Myst2 HAT complex Niam participates in. Affinity purification in combination with blue native polyacrylamide gel electrophoresis followed by protein correlation profiling mass spectrometry (ABC-MS) has proven useful in resolving alternative protein complexes containing shared subunits^24, 30^. To address which Myst2 HAT complex contains Niam, we purified Myst2 complexes by single FLAG immunoprecipitation and fractionated the native eluate by BN-PAGE, excised the gel lane in 48 fractions, analysed them by quantitative mass spectrometry and performed hierarchical clustering of the migration profiles (Fig. 3d). Niam clustered closely with Ing4/5 and JADE proteins Phf15/16/17, but not with Brpf3 and Brd1. These results further suggest that in mouse ESCs Niam might be part of a JADE-containing Myst2 H4 HAT complex.

### Acetylation of Myst2-associated proteins

In addition to acetylating histones, Myst2 has also been shown to acetylate other non-histone substrates^3^. We therefore looked for acetylated peptides in Myst2-associated proteins by searching the mass spectrometry data against the protein database using lysine and arginine acetylation as variable modifications. Sixteen acetylated peptides were identified representing six unique sequences in five proteins (Table 1). Histone acetylase complex members Brpf3 (Lys-1038) and Ing4 (Lys-127 and Lys-129) were found to be acetylated. It is well known that some HAT complex members are acetylated *in vivo* ^3^. Ing4 has previously been shown to be acetylated at these sites, whilst Brpf3 has previously been shown to be acetylated at other sites^31–33^, but not the one described here. We also detected acetylated peptides of histone H3 at positions Lys-18 and Lys-23, two well-known acetylation marks of histone H3.

**Table 1:** Acetylated peptides from Myst2-associated proteins.

Protein name	Evidence peptide	Site	Novel
Niam	R.AA**K**ATVFENASICDEIAR.L + Acetyl (K)	K45	Yes
	M.**S**VLSGLASEPRTPLSS**K**AR.M + Acetyl (K); Acetyl (Protein N-term)	K18, S2	Yes, Yes
Ing4	K.QIESSDYDSSSS**K**G**K**K.S + 2 Acetyl (K)	K127, K129	No, No
Brpf3	R.G**K**PALSRVPFLEGVNGDSDHSGSGR.S + Acetyl (K)	K1038	Yes
Histone H3	R.**K**QLAT**K**AAR.K + 2 Acetyl (K)	K18, K23	Yes, Yes

Novel peptides are those not previously reported in the literature. Modified residues are shown in bold.

Novel interactor Niam was found to be acetylated at two positions, Lys-18 and Lys-45, in addition to carrying N-terminal acetylation (Table 1). These modifications were found both in Myst2 TAP-MS and Niam AP-MS experiments (see below). This protein has never before been detected in acetylation screens, possibly due to its low abundance in somatic cells. The two acetylated lysines lay in a low complexity region rich in charged amino acids, probably a region of disordered structure, which is suggested to be involved in protein-protein interactions^34, 35^. Our results raised the possibility that Myst2 could be responsible for Niam acetylation. We tested this hypothesis by performing *in vitro* acetylation assays with recombinant Myst2 and Niam proteins. However, we have so far been unable to show that this is the case (Supplementary Fig. S2).

### Niam associates with chromatin remodelling and cell proliferation protein assemblies

**Figure 4:**
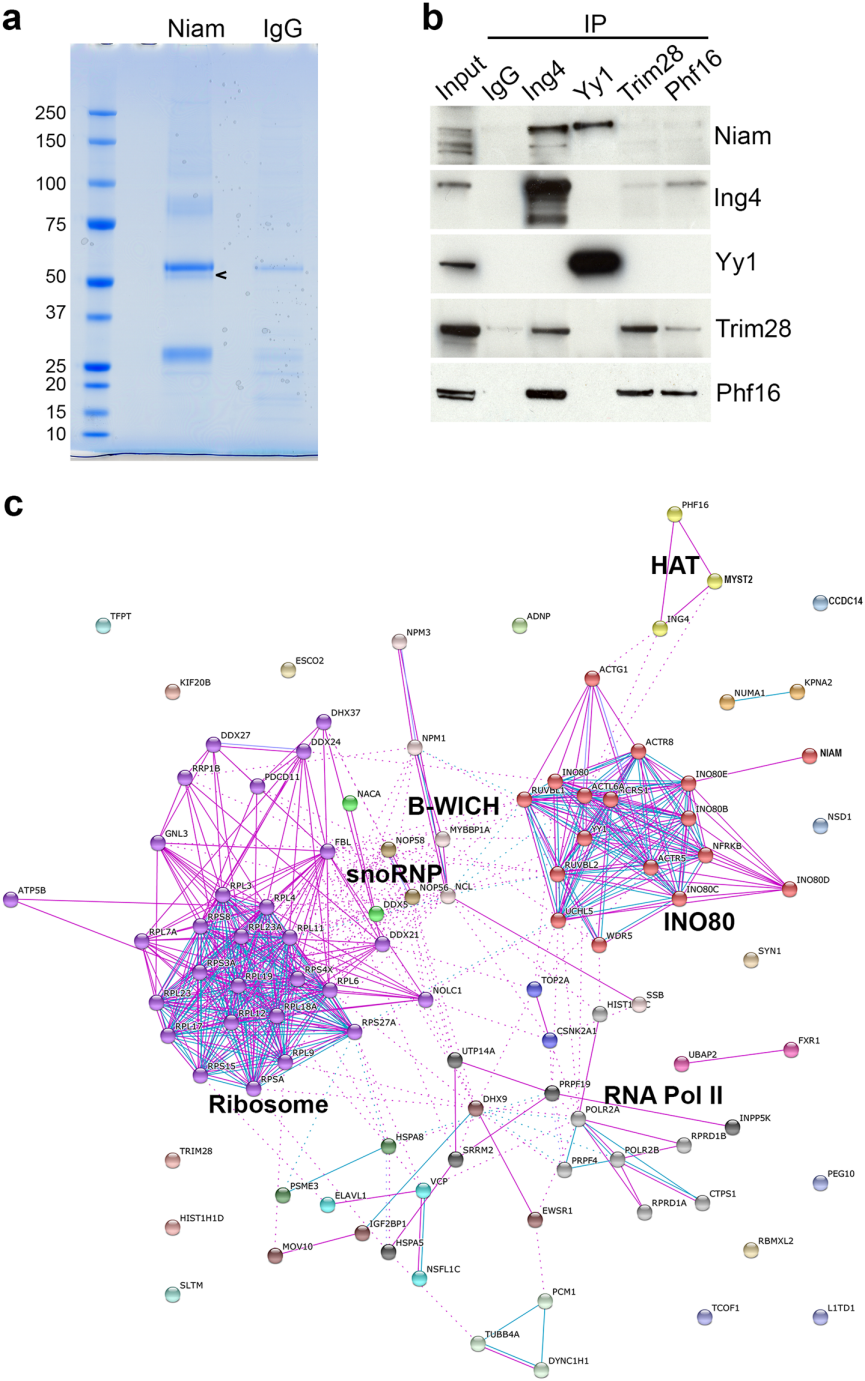
Identification of Niam binding proteins. (**a**) Coomassie-stained gel of proteins immunoprecipitated with anti-Niam antibody and control IgG in mouse ESCs. The arrow head indicates Niam. (**b**) Validation of Niam interactions by reverse co-immunoprecipitation. Full-length blots are presented in Supplementary Fig. S5. (**c**) Network of interactions between Niam co-immunoprecipitating proteins as obtained from STRING clustered with the MCL algorithm. Pink edges denote interactions from interaction databases and blue edges denote interactions from curated databases. Dotted lines represent inter-cluster edges.

To expand our understanding of Niam we performed large-scale immunoprecipitation of endogenous Niam complexes and identified its associated proteins by mass spectrometry in three replicate experiments (Fig. 4a). Niam was conclusively identified in all three, with an average of 9 unique matching peptides. We also identified 102 putative Niam partners by one or more high confidence peptides in all three replicates (Supplementary Table S2). Myst2 was amongst them (average of 10 unique peptides), further confirming its interaction with Niam. Other members of Myst2 H4 HAT complexes such as Ing4 and Phf16 were also identified. Phf17, by contrast, was not found in the dataset. Meaf6, a shared component of both Myst2 H3 and H4 HAT complexes, and Brpf subunits of Myst2 H3 HAT complexes were not detected either. These results suggest that Niam is part of a Myst2 sub-complex containing at least a JADE subunit (and no Meaf6).

In order to confirm some of the Niam interactions we carried out reverse co-immunoprecipitation experiments using selected Niam interactors as baits, namely Yy1, Ing4, Trim28 and Phf16, and tested for the presence of Niam by western blot. Immunoprecipitation with antibodies against endogenous Yy1 and Ing4 pulled down the intended baits and Niam, confirming that Niam associates with these two proteins in mouse embryonic stem cells (Fig. 4b). Trim28 was found in the Ing4 and Phf16 immunoprecipitate, and vice versa, Ing4 and Phf16 were found in the Trim28 immunoprecipitate. This indicates that Trim28 interacts with Ing4 and Phf16, and suggests that its interaction with Niam might be mediated by these two proteins. These results show that this set of proteins are intimately linked and suggest that some of their interactions with Niam might be indirect.

To dissect the list of proteins, GO terms were annotated using DAVID Bioinformatics Resources tool^36^ (Supplementary Table S3). In the cellular compartment category, over 80% of genes were annotated with the term nucleus. More specifically, there was a strong enrichment in genes localised to nucleolus, consistent with the nucleolar localisation of Niam^28^. The terms ribosome and chromosome were also enriched, as were linked biological processes such as ribosome biogenesis, translation, DNA repair, and cellular response to DNA damage. This finding is in agreement with NIAM’s established role in proliferation^28, 29, 37^, its induction by DNA damage^37^, and the well described roles of NIAM’s partner, ARF, in ribosome biogenesis and translation (reviewed in^38^). Chromatin remodelling and transcription were also enriched terms, possibly reflecting cellular roles associated to Niam’s interaction with the Myst2 HAT complex. As for molecular function, poly(A) RNA binding was the most enriched term, together with ribosome structural component, helicase activity and transcription factor binding. This suggests that Niam could be involved in various processes by means of interaction with distinct protein assemblies.

In order to take a global view, we retrieved interactions between all Niam preys using STRING database^39^ and displayed the resulting network (Fig. 4c). Clustering of the nodes revealed distinct molecular machines where several or all subunits had been identified as Niam preys. These included the INO80 complex, the ribosome, the RNA polymerase II complex, the B-WICH complex and the snoRNP complex amongst others. The high degree of previously described interactions between Niam preys provides additional support to their identification as *bona fide* binding partners. All these clusters are connected between them, reflecting the closeness of these machineries and the processes they are involved in. The INO80 complex is a chromatin remodelling complex involved in transcription, DNA replication and repair^40–42^. All reported subunits of this complex were identified with high protein scores, suggesting it interacts with Niam strongly and directly (Supplementary Table S2). Our results confirm the finding from a large scale human interactome study where INO80E was identified as a NIAM-interacting protein by yeast two-hybrid^43^. The B-WICH complex is a chromatin remodelling machine that is also involved in DNA replication in addition to rDNA transcription^44^. This is interesting in light of the role of Myst2 in replication^21, 22, 45^. Our data strongly suggest that Niam might also have roles in transcription, DNA replication and repair, cooperating with Myst2.

### A role for the Myst2 and Niam interaction network in development and disease

**Figure 5:**
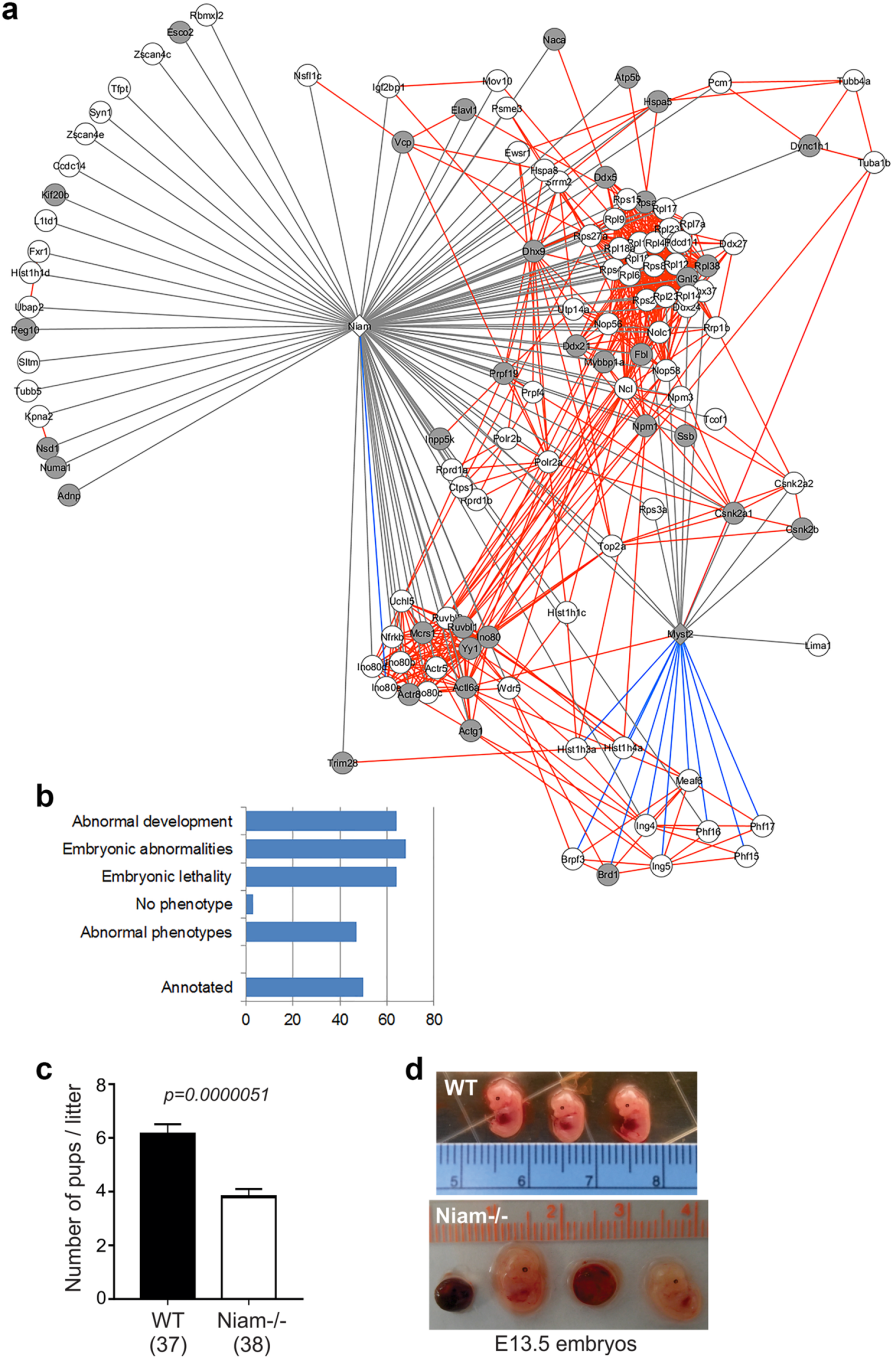
Extended Myst2-Niam protein interactome and role for Niam in embryonic development. (**a**) Myst2 and Niam are shown as diamonds. Grey edges represent interactions observed experimentally in our study. Red edges represent known physical interactions retrieved from STRING and literature. Blue edges represent interactions observed in this study that had previously been reported. Light grey nodes are genes whose loss-of-function results in embryonic lethality. (**b**) Distribution of loss of function phenotypes. Numbers shown are percentage of genes with MGI annotation. (**c**) Litter sizes at birth for wild-type (WT) and *Niam*−/− mice. The number of litters studied is indicated in parentheses, and results are presented as the mean +/− sem. Statistical significance was determined by an unpaired 2-tailed Student *t*-test (*p* = 0.0000051). (**d**) Comparative images of embryos isolated from WT or *Niam*−/− pregnant females at embryonic day 13.5 (E13.5).

Myst2 is essential for mammalian development, its absence resulting in embryonic lethality^6^. NIAM functions as an anti-proliferative factor in somatic cells and is required for maintaining chromosomal stability, suggesting NIAM may act as a tumour suppressor^28, 29^. In support of that conclusion, NIAM expression is down-regulated by oncogenic miR-155 in B-cell lymphoma^46^ and Niam-deficient mice develop spontaneous tumours, most notably B-cell lymphoma^37^. To date, a possible role for Niam in embryonic biology has not been investigated, although it was first identified as a TGFβ-regulated gene in preimplantation mouse embryos, hence its original designation as Tbrg1^47^. We therefore explored whether the Myst2-Niam network is generally involved in embryonic development. The MGI database contained annotations for 59 out of 118 input genes (Supplementary Table S4). Loss-of-function abnormal phenotypes were reported for 56 proteins (47% of the network, 56/118). 68% (38/56) showed embryonic phenotypes, with 64% (36/56) resulting in embryonic lethality (Fig. 5a,b). In addition, 64% (36/56) displayed abnormal development phenotypes. Based on those findings we carefully monitored litter sizes in Niam-deficient mice versus wild-type counterparts. As shown in Fig. 5c, *Niam*−/− females bred to *Niam*−/− male mice had significantly smaller litter sizes (average of 3.8 pups per litter) compared to wild-type mouse breedings (average of 6.1 pups per litter). Notably, embryos isolated from *Niam*−/− mice at embryonic day 13.5 displayed greater incidence of embryonic lethality and other developmental abnormalities, such as increased size, compared to wild-type embryos (Fig. 5d). These results strongly suggest that the Myst2-Niam protein interaction network is vital in early stages of development in the mouse.

MYST2 has been linked to cancer by way of its role in replication and also through is interaction with the androgen receptor and tumour suppressors ING4 and ING5^1^. Some studies point to *MYST2* being an oncogene, since its overexpression enhances the transformed phenotype of cancer cells^48^. Furthermore, MYST2 is overexpressed in several cancer tissues^49^. In contrast, NIAM mRNA and protein expression are markedly down-regulated in numerous cancers and *Niam* mutant mice expressing low levels of the protein develop spontaneous tumours^28, 37, 46^. Given the involvement of both proteins in cancer, we asked whether human orthologues of the Myst2-Niam network are responsible for the development of cancer. We queried the Cancer Gene Census database and found that somatic mutations in eight human orthologues of Niam-Myst2 network proteins are causally linked to cancer (Supplementary Table S5). We also investigated their involvement in hereditary diseases by querying the OMIM database. Sixteen genes encoding orthologues of nodes of the Niam-Myst2 network are involved in human genetic disease (Supplementary Table S5).

These results further stress the importance of Myst2 and Niam in development and carcinogenesis, and provide a list of priority candidate genes to investigate in depth the mechanisms by which these proteins contribute to disease.

## Discussion

In this study we have undertaken the identification of Myst2 protein interactions in mouse embryonic stem cells. We have described the existence of Myst2 H3 and H4 HAT complexes in mouse ESCs for the first time. Furthermore, we have discovered a novel Myst2 interactor, Niam, a minimally characterised protein with established tumour suppressive activity in somatic cells and mice^28, 29, 37, 46^. We have also performed the first comprehensive proteomic study of Niam interactions.

Our results show that in mouse ESCs Myst2 is part of MYST2-JADE-type and MYST2-BRPF-type HAT complexes, in a fashion similar to somatic cells^7, 9, 11, 12, 14^. Jade1/Phf17 is the most abundant subunit of MYST-ING-JADE complexes, followed by Phf16 and Phf15. Brpf3 seems to be the main BRPF subunit in Myst2 H3 HAT complexes in mouse ESCs, and Brpf2/Brd1 also participates in them, albeit at much lower level. Whether each type has independent functions or targets different sets of genes is currently unknown, but at least in somatic cells Brpf3 is required for DNA replication^14^ whilst Brpf1 and Brpf2 are not^12^. Intriguingly, whilst lack of the latter results in embryonic lethality, absence of Brpf3 does not^14^. In agreement with this, although Myst2 is required for replication in somatic cells, it seems not to be essential for replication in the embryo^6^.

Our search for novel interactors of Myst2 identified Niam as a robust binding partner. The presence of acetylated residues on Niam suggested it could be a novel substrate of Myst2. However, we have been unable to demonstrate this *in vitro*, hinting that this might not be the case. Although we did not identify other acetyltransferases co-purifying with Niam, suggesting than in ESCs Myst2 is the major acetyltransferase interacting with Niam, another acetyltransferase might be responsible. Indeed NIAM interacts with the MYST family member TIP60 in cancer cells^29^ and we have preliminary data indicating that Myst2 interacts with Tip60 in mouse ESCs. Alternatively, additional factors might be required for Niam acetylation, which would suggest that acetylation is tightly regulated *in vivo*. The strength and abundance of the interaction between Niam and Myst2 insinuates that it has functions other than an enzymatic reaction. Niam also interacts with other HAT subunits, at least one representative of the JADE family, Phf16, and one ING protein, Ing4. We did not find Ing5 and Meaf6, the fourth component of Myst2 HAT complexes, amongst Niam preys, suggesting either that Niam might not interact with full tetrameric Myst2 H4 HAT complexes or that our assay is not sensitive enough to detect them. Interestingly, Phf16 does not enhance H4 acetylation by Myst2 like Phf17 does^17^, arguing these two family members are not functionally redundant. It is possible that a Niam-Myst2-Phf16-Ing4 complex has a role unrelated to histone H4 acetylation in ESCs. Notably, in cancer cells NIAM cooperates with TIP60 to activate p53^29^. The fact that Myst2 HAT complexes have been extensively studied in somatic cells and no Niam has ever been identified, suggests that the Niam-Myst2-Phf16-Ing4 complex has an ESC specific role.

Mouse Niam is a member of the F or Y-rich N-terminus and C-terminus containing family. This domain of unknown function occurs frequently in different chromatin-associated proteins, and is particularly common in histone demethylases like trithorax and MLL proteins^50^. Indeed Niam was recently shown to associate with chromatin, although the FY rich domains that reside in the Niam C-terminus are not required for chromatin binding^29^. Consistent with that observation, our *in vitro* binding assays demonstrated that the amino terminus of Niam, which mediates nearly all of Niam’s known functions, is necessary and sufficient for association with endogenous Myst2 in mESC lysates. Niam is conserved in metazoans and seems to have evolved alongside the p53/MDM2 pathway components^50^. Rat Niam has a region of low similarity to a region of mouse Mll2 which plays a role in protein complex assembly^50^. Interestingly the worm *Caenorhabditis elegans* has only one FY-rich domain protein, SET-16. This protein also contains a HMG domain (DNA binding), a SET domain (histone binding, methyltransferase) and strikingly, many PHD domains (responsible for binding to histones) which are also present in Jade and Brpf proteins and direct the specificity of Myst2 HAT activity towards histone tails^11^. This raises the possibility that Niam’s chromatin association is facilitated through interaction with its PHD domain-containing partners Phf16 and Ing4.

Both Myst2 and Niam have been shown to have a role in proliferation of somatic cells and numerous connections to p53. MYST2 associates with p53 in MCF7 cells, where p53 regulates its HAT activity^51^. Knockdown of *MYST2* in Hela cells results in down-regulation of multiple p53-regulated genes^16^. NIAM binds to p53 regulators ARF, MDM2, and TIP60, and through those interactions stabilizes and activates p53^28, 37^. Our analysis did not identify p53 or its key regulators among Niam partners in mESCs. This is consistent with the fact that in ESCs ARF is tightly silenced^52^ and p53, although highly abundant, is mostly restricted to the cytoplasm^53–55^, where its ability to transcribe targets including MDM2 would be limited. Indeed, p53 activity appears constrained in ESCs since they are largely unaffected by stress-induced (e.g. DNA damage) p53-mediated signals^55, 56^. It is therefore not surprising that we found high levels of Niam protein in mouse ESCs, since earlier work in somatic cells showed that functional p53 restricts NIAM expression^28^. Elevated Niam levels in ESCs may also result from transcriptional upregulation by pluripotency factors Oct4 and Nanog among others, which have been shown to target *Niam* (evidence from ESCAPE database^57^).

In mouse embryos lack of Myst2 seems to have no effect on cell proliferation, in contrast with reports in human cancer cell lines, but results in transcription defects which cause embryonic lethality^6^. By comparison, we show here that Niam loss in mice causes reduced litter sizes that reflect problems during embryonic development, including altered size and lethality of the embryos. As we previously reported, Niam-deficient embryos that survive through birth display no post-natal defects in viability, but have enhanced tumour susceptibility^37^. The data presented here strongly suggest that in ESCs Niam may have yet undiscovered roles in transcription, DNA replication and/or repair, mediated by its interaction with Myst2 and various other chromatin remodelling complexes, namely INO80 and B-WICH complexes. The interactions of Niam with Myst2 and the INO80 and B-WICH complexes seem to be mutually exclusive, since we did not identify members of these complexes as Myst2 preys. Loss of Niam in somatic cells results in abnormal chromosomal segregation and causes aneuploidy, a major hallmark of advanced cancer. Interestingly, the INO80 complex, which seems to be a close Niam interactor, is also involved in chromosome segregation through its binding to microtubules and its requirement for microtubule assembly during mitosis^41^. Niam also interacts with tubulin, raising the possibility that it might cooperate with INO80 in this role.

MYST2 interacts with replication factors ORC1 and MCM2^20, 58^ and binds to replication origins^22, 59^. We did not identify any pre-replicative complex proteins as Myst2 partners in our TAP-MS study. The different experimental conditions or the sensitivity of our assay could explain this. Another possibility is that Myst2 does not interact with the pre-replicative complex in ESCs. Myst2 knockout mice do not display replication defects, and therefore it has been suggested that Myst2 is not essential for replication in the embryo^6^. Interestingly, in human cancer cell lines MYST2 levels at replication origins are ten times those of normal fibroblasts^49^. Ing proteins do not associate with Myst2 at origins of replication^22^ but Jade-1/Phf17 in cooperation with Myst2 enhances MCM loading^45^ and it has been suggested that Jade promotes precise targeting of Myst2 to origins of replication^18^. This raises the question of whether Niam localises at origins of replication, as also do members of the INO80^60^ and B-WICH complexes^61^. Over-expression of NIAM induces a cell cycle block in G1^28^, suggesting that it might inhibit DNA replication. The INO80 complex also has multiple roles in DNA repair and replication^40, 41^. INO80 binds to replication forks during S phase and promotes their progression, and its absence in the mouse embryo leads to defects in DNA replication that ultimately result in embryonic lethality^42^. The B-WICH complex is also recruited to replication foci during DNA replication and has a role in the maintenance of chromatin structures^62, 63^.

The B-WICH complex also facilitates the recruitment of HATs to rDNA promoters, most likely through another factor^64^. Although Myst2 has not been reported in the regulation of rDNA gene expression, yeast MYST HAT Esa1, which is mostly responsible for H4 acetylation, associates with nucleolar rDNA and is involved in its transcriptional silencing^65^. Niam has abundant links to nucleolar proteins, also hinting at a potential nucleolar role. Whether this would be in association with Myst2 or not is unknown, although it seems unlikely since Myst2 seems to be excluded from the nucleolus (Human Protein Atlas).

In summary, we have shown here for the first time that Myst2 is part of histone H3 and H4 acetylation complexes containing Brpf and Jade subunits in mouse ESCs, in a similar manner to somatic cells. We have identified a novel Myst2 binding partner, the tumour-suppressor protein Niam. We have also expanded the molecular context around Niam by describing a set of associated proteins, which include transcription, DNA replication/repair and ribosome biogenesis and splicing machineries. These results not only uncover a new fairly uncharacterised protein that might impinge on Myst2 function, but also suggest novel embryonic functions for Niam and strongly argue for previously unrecognised roles for this protein, particularly in DNA replication, repair and transcription. These data should provide an excellent basis for investigating mechanistic aspects of the roles of Myst2 and Niam in a variety of essential cellular functions, and therefore contribute to increased understanding of their role in pluripotency and organism early development, and in the development of cancer.

## Methods

### Targeting vector construction

Gene targeting vectors were constructed by a two-step recombineering process from 129S5 strain mouse genomic BAC clones identified using the Ensembl genome browser. BAC cultures were transformed with the pSC101-BAD-gbA plasmid^66^. Transformants were selected for growth in tetracycline (5 μg/ml) at 30 °C. DNA sequences corresponding to the entire open reading frame of the affinity tag were PCR amplified from a plasmid template with a high fidelity enzyme (Fast Start High Fidelity kit, Roche) and using oligonucleotide primers consisting of 50 bp of gene-specific sequence appended to 20 bp of sequence complementary to the tag (Supplementary Fig. S1). Amplification products were gel purified, digested with *Dpn*I to eliminate the original template, and microdialysed against water prior to recombineering. To introduce sequences encoding the epitope tag into the BAC clone by recombineering, expression of the necessary proteins was induced by addition of arabinose to the growth medium (0.2%, Sigma) and increasing the temperature to 37 °C for a brief period (40 minutes) in a culture of cells carrying both the appropriate BAC and the recombineering plasmid. The cells were then cooled, made electrocompetent by repeated washing in cold HPLC grade water (HiPerSolv, BDH), and electroporated with 1–2 μg of purified PCR product. Recombinants were selected by growth in kanamycin (15 μg/ml), chloramphenicol (12.5 μg/ml) and tetracycline (5 μg/ml). A second gap repair step retrieved modified BAC sequence into a plasmid backbone^67^ to create a targeting vector, following selection for growth in both kanamycin (15 μg/ml) and ampicillin (50 μg/ml). The final vector was verified by sequencing across the junction of genomic sequence and the tag and through the entirety of the epitope tag prior to introduction into ESCs.

### ES cell culture

The feeder-independent 129ola strain derived ESC line, E14Tg2a, was used for all gene targeting experiments. Cells were cultured on 0.1% gelatin tissue culture plates and maintained in GMEM (Sigma) supplemented with 2 mM glutamine, 100 μM β-mercaptoethanol, 10% fetal calf serum (Invitrogen) and 500 U/ml leukaemia-inhibitory factor (ESGRO, Millipore). Gene targeting vectors were introduced by electroporation^68^. 3–5 × 10e7 cells were electroporated with 25 μg of vector DNA, linearised with *AsiS*I, precipitated and resuspended in sterile PBS. Stably transfected ES cell clones were selected by growth in ESC medium containing 175 μg/ml G418 (Invitrogen), expanded and frozen in liquid nitrogen.

### Genotyping

ES cell clones containing a targeted insertion of the *Myst2*-FTAP tag were identified by long range PCR amplification of genomic DNA. Genomic DNA was prepared for genotyping as described previously^68^ and approximately 500 ng of DNA were subjected to long range PCR amplification (Expand Long Template, Roche) according to manufacturer’s specifications, using a combination of locus and tag-specific primers (see Supplementary Fig. 1a, primer sequences on request). Whole cell extracts derived from a selection of PCR positive clones were also subjected to Western blotting to verify expression of the predicted fusion protein using an anti-FLAG antibody (anti-FLAG M2, Sigma).

### Affinity purification

Feeder-free E14 murine embryonic stem cell (ESC) lines expressing Myst2-FTAP were used for affinity purification of Myst2, and an E14 mouse ESC line expressing a beta-gal-FTAP fusion protein (created by random integration of a gene trap vector harbouring the tagging cassette into an unknown ORF expressed in ES cells) was used as control. Whole cell extracts were prepared from 10e9 cells using a high salt lysis buffer (450 mM NaCl) and affinity purifications performed as previously described^23^. For tandem affinity purifications, the TEV eluate was diluted with calmodulin binding buffer (CBB: 10 mM Tris-HCl pH 8, 150 mM NaCl, 0.1% NP-40, 1 mM magnesium acetate, 1 mM imidazole, 2 mM calcium chloride, 1 mM DTT) and then incubated with calmodulin resin (Stratagene) for 60 min at 4 °C. Final elution was carried out in CBB containing EGTA instead of calcium chloride. EGTA eluates were concentrated in Vivaspin 500 PES centrifugal filters (Vivascience), reduced with 1 mM DTT and alkylated with 2 mM iodoacetamide prior to sample fractionation by polyacrylamide gel electrophoresis using Novex NuPAGE Bis-Tris 4–12% gels (Invitrogen). Gels were stained with colloidal Coomassie (Sigma). Whole lanes were cut in approximately 24 slices, gel pieces were de-stained completely and digested with trypsin (sequencing grade, Roche). Peptides were extracted using 0.25% formic acid-50% acetonitrile and dried in a Speed Vac (Thermo).

For co-immunoprecipitation experiments, antibodies (2–5 μg) were coupled to Dynal-Protein G beads. Antibody-coupled beads were incubated with whole cell extracts for 1–2 h at 4 °C. Immunoprecipitated proteins were eluted by incubating the beads in LDS sample loading buffer at 70 °C for 10 min. Input and eluted samples were analysed by western blot. The following antibodies were used: monoclonal and polyclonal anti-Niam^28, 69^, anti-FLAG M2 (Sigma), anti-Myst2 (ab70183, Abcam), anti-Phf17 (sc-160450, Santa Cruz Biotechnology), anti-Ing5 (ab3716, Abcam), anti-Ing4 (ab108621, Abcam), anti-Yy1 (ab109237, Abcam), anti-Kap1 (ab10484, Abcam), anti-Trim28 (ab10484, Abcam), anti-Phf16 (ab129495, Abcam).

### *In vitro* binding assays

Bacterial glutathione S-transferase (GST)-tagged fusions proteins for mouse and human Niam were prepared and purified as described previously^28^. Mouse ESC pellets were lysed on ice in NP-40 buffer (50 mM Tris [pH 8.0], 120 mM NaCl, 1 mM EDTA, 0.5% Nonidet P-40, and 0.1 mM Na3VO4) supplemented with 1 mM NaF, 30 uM phenylmethylsulfonyl fluoride (PMSF), protease inhibitor cocktail (Sigma) and phosphatase inhibitor cocktail (Sigma). Lysates were sonicated and clarified by centrifugation (15,000 rpm for 10 min) at 4 °C, and 0.5 to 1 mg of protein added to binding reactions containing purified GST or GST-Niam proteins previously bound to glutathione sepharose. Reactions were incubated overnight at 4 °C on a rotator and the next day complexes were washed 4 times with ice-cold NP-40 buffer and resolved by SDS-PAGE. Proteins were transferred onto PVDF (Millipore) membranes and the levels of GST proteins in each reaction measured by Ponceau S staining. Binding of endogenous Myst2 was detected by immunoblotting with the anti-Myst2 antibody (ab70813, Abcam).

### Blue native PAGE

Sample preparation and fractionation by blue native PAGE was carried out from 5 × 10e8 cells as previously described^24, 30^.

### Mass spectrometry and data analysis

Peptides were re-dissolved in 0.5% formic acid and analysed with on-line nano liquid chromatography tandem mass spectrometry on an LTQ FT (Thermo Fisher Scientific), as previously described^23^.

The raw files were processed with Proteome Discoverer v1.3 and v1.4 (Thermo). Database searches were performed with Mascot (Matrix Science) against the mouse Uniprot database (v. February 2013 and January 2015). The search parameters were: trypsin digestion, 2 missed cleavages, 10 ppm mass tolerance for MS, 0.5 Da mass tolerance for MS/MS, with variable modifications of carbamidomethyl (C), N-acetylation (protein), oxidation (M), and pyro-glu (N-term Q). Database search results were refined through processing with Percolator (significance threshold <0.05, FDR <1%). High confidence peptides were apportioned to proteins using Mascot Protein Family summary. Protein identification required at least one high-confidence peptide (FDR <1%).

External contaminants (keratins, albumin, casein, trypsin, TEV protease) were removed from protein lists. We report only proteins identified by more than two high confidence peptides in at least one of the replicates and have chosen one representative of each set of proteins identified by the same peptides (meaningful gene symbol and highest molecular weight). Protein lists for target and control purifications were compared and high confidence true interactors identified as previously described^23^.

Processing of mass spectrometry raw data from blue native PAGE experiments for protein correlation profiling was performed as previously described^24, 30^.

The mass spectrometry proteomics data have been deposited to the ProteomeXchange Consortium via the PRIDE^70^ partner repository with the dataset identifier PXD005987.

### Mouse studies

*Niam*-deficient mice (C57BL/6 strain) were generated through the Knockout Mouse Project (KOMP) and have been previously described^37^. Mice were housed in the University of Iowa Animal Care Facility, and all mouse experiments were conducted according to approved protocols by the University of Iowa Animal Care and Use Committee. The number of pups per litter was determined at weaning following wild-type (WT) mouse breedings or *Niam*−/− mouse breedings, such that all offspring within each litter were of identical genotype and homozygous for the *Niam* allele (either +/+ or −/−). Genotypes of the breeders were confirmed by PCR of tail genomic DNA^37^. Embryos were isolated for analysis from pregnant females of each genotype at day 13.5 *post coitum*. Briefly, uterine horns were removed and placed into petri dishes containing PBS, and each embryo separated manually from its placenta and embryonic sac prior to imaging.

### Bioinformatic analyses

Enriched GO terms and functional clusters were retrieved using DAVID 6.8 database^36^. Protein-protein interactions between Niam preys were retrieved using STRING database v. 9.1, with predicted functional links derived from experiments and curated databases at medium confidence level. Nodes were clustered using MCL algorithm at inflation 2. Mammalian phenotype ontology annotations were retrieved from the Mouse Genome Informatics (MGI) database (v. 6.07)^71^. Human disease associations were obtained from OMIM^72^ through Ensembl Biomart. Genes with known cancer causing mutations were obtained from the Cancer Gene Census^73^.

## Electronic supplementary material

Supplementary Information

Supplementary Table S3

Supplementary Table S4
