# Supplementary material for: Myst2/Kat7 histone acetyltransferase interaction proteomics reveals tumour-suppressor Niam as a novel binding partner in embryonic stem cells
Source: Sci Rep. 2017 Aug 15;7:8157. doi: 10.1038/s41598-017-08456-2 (PMC5557939; doi:10.1038/s41598-017-08456-2)
Supplement: Supplementary file 1 — Supplementary Information [file 41598_2017_8456_MOESM1_ESM.pdf]

Supplementary Information

Myst2/Kat7 histone acetyltransferase interaction proteomics reveals tumour-suppressor Niam as a novel binding partner in embryonic stem cells

Mercedes Pardo[1,*], Lu Yu[1], Shih Pei Shen[2], Peri Tate[2], Daniel Bode[1], Blake L. Letney[3], Dawn E. Quelle[3], William Skarnes[2], and Jyoti S. Choudhary[1]

[1] Proteomic Mass Spectrometry, Wellcome Trust Sanger Institute, Cambridge, Cambridgeshire, United Kingdom

[2] Stem Cell Engineering, Wellcome Trust Sanger Institute, Cambridge, Cambridgeshire, United Kingdom

[3] Departments of Pharmacology and Pathology, The University of Iowa and Holden Comprehensive Cancer Center, Iowa City, IA, 52242, USA

* Corresponding author: Mercedes Pardo (mp3@sanger.ac.uk)

Supplementary Figure S1

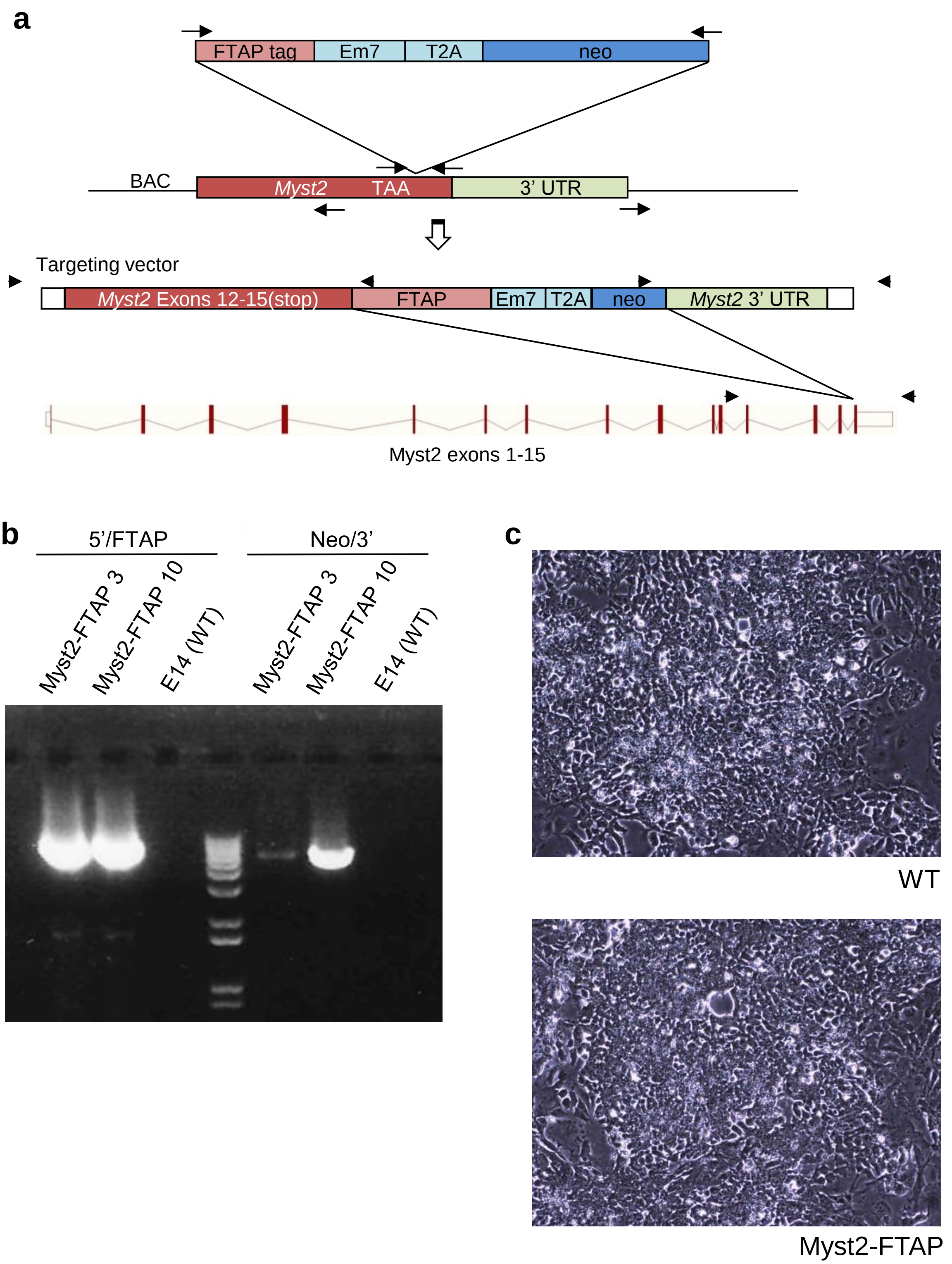

Supplementary Figure S1. Targeted tagging of the *Myst2* locus. a. The epitope tag and antibiotic resistance cassette was introduced into a *Myst2*-containing BAC clone by recombineering. A gene targeting vector with 5 kb of target gene homology flanking either end of the tag was then generated from the modified BAC by gap repair. Arrows indicated the location of gene-specific primer sequences used for recombineering. **b.** Correctly targeted ES cell clones were identified by long range PCR amplification of genomic DNA using a combination of locus-specific and cassette-specific primers (arrows in panel **a**). Two representative clones (#3 and #10) and wild type E14 cells are shown. Clone 10 was used for further studies. **c**. Representative images of wild type E14 and Myst2-FTAP cell cultures.

Supplementary Figure S2

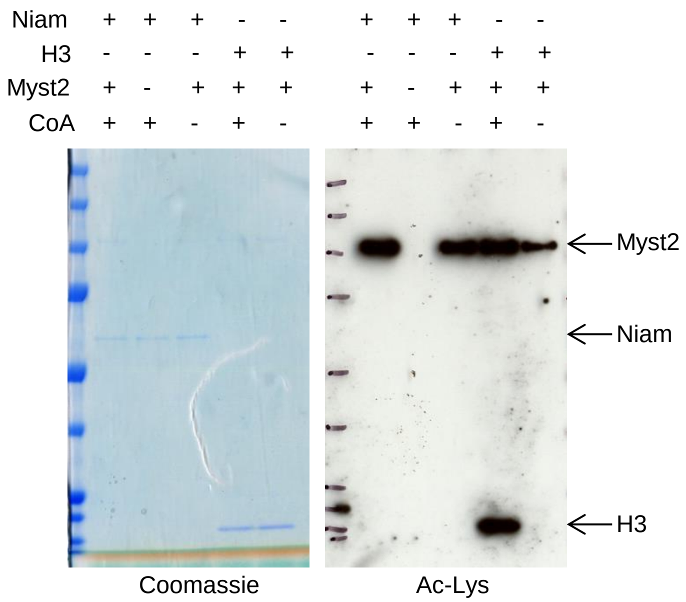

Supplementary Figure S2. Myst2 acetyltransferase assay. Commercially available purified proteins were employed in the acetyltransferase assay. Myst2 was incubated with Niam or histone H3 in acetyltransferase buffer in the presence of acetyl-CoA. After 60 min samples were separated by SDS-PAGE and analysed by western blotting with an antibody against pan-lysine acetylation (Cell Signalling Technology). Myst2 was found to auto-acetylate, as previously described. We did not detect acetylation of Niam by Western blot.

Supplementary Figure S3

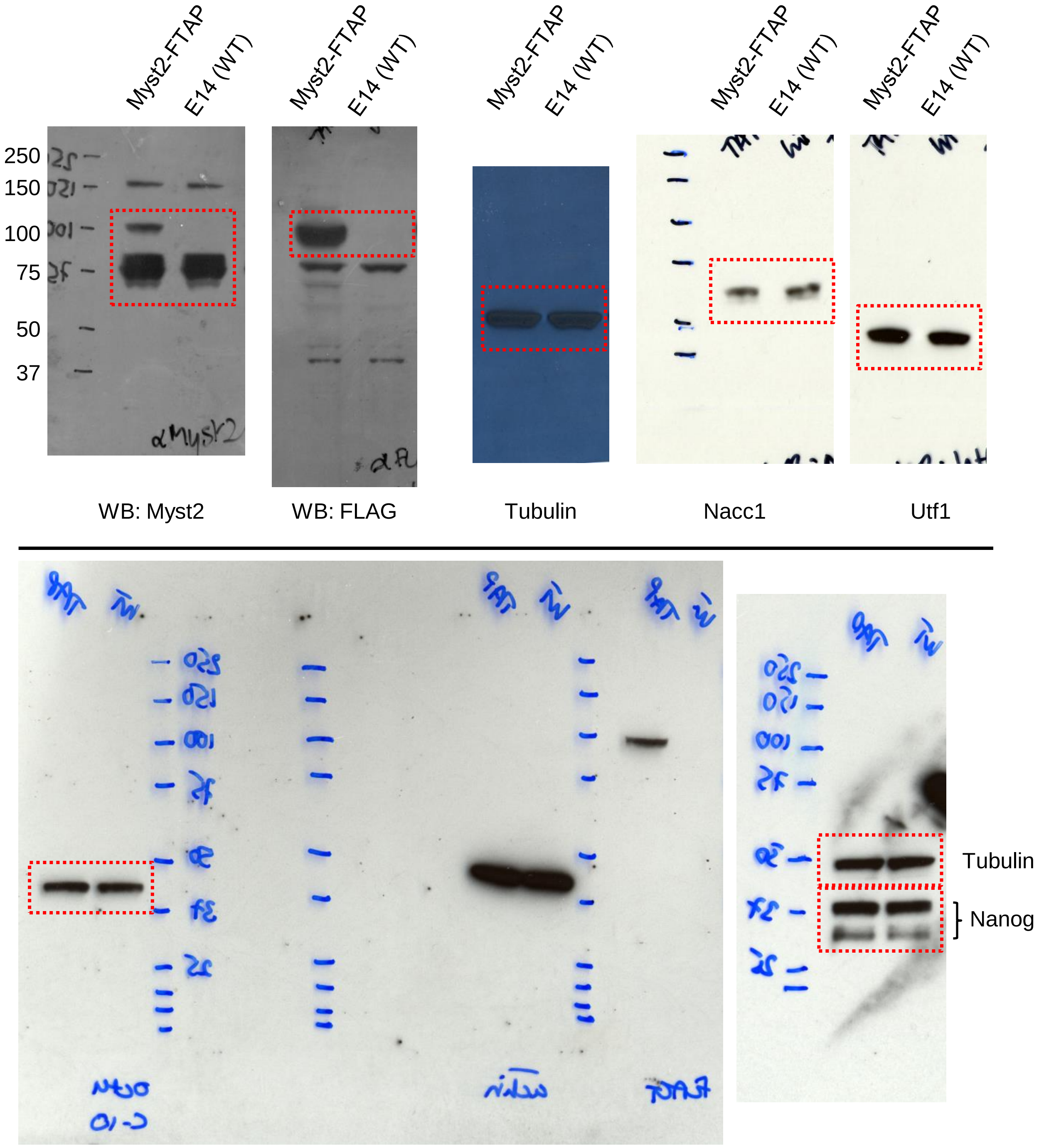

Supplementary Figure S3. Full-length images of western blots presented in Fig. 2a. The cropped images shown in Fig. 2a are marked here with a red dotted line.

Supplementary Figure S4

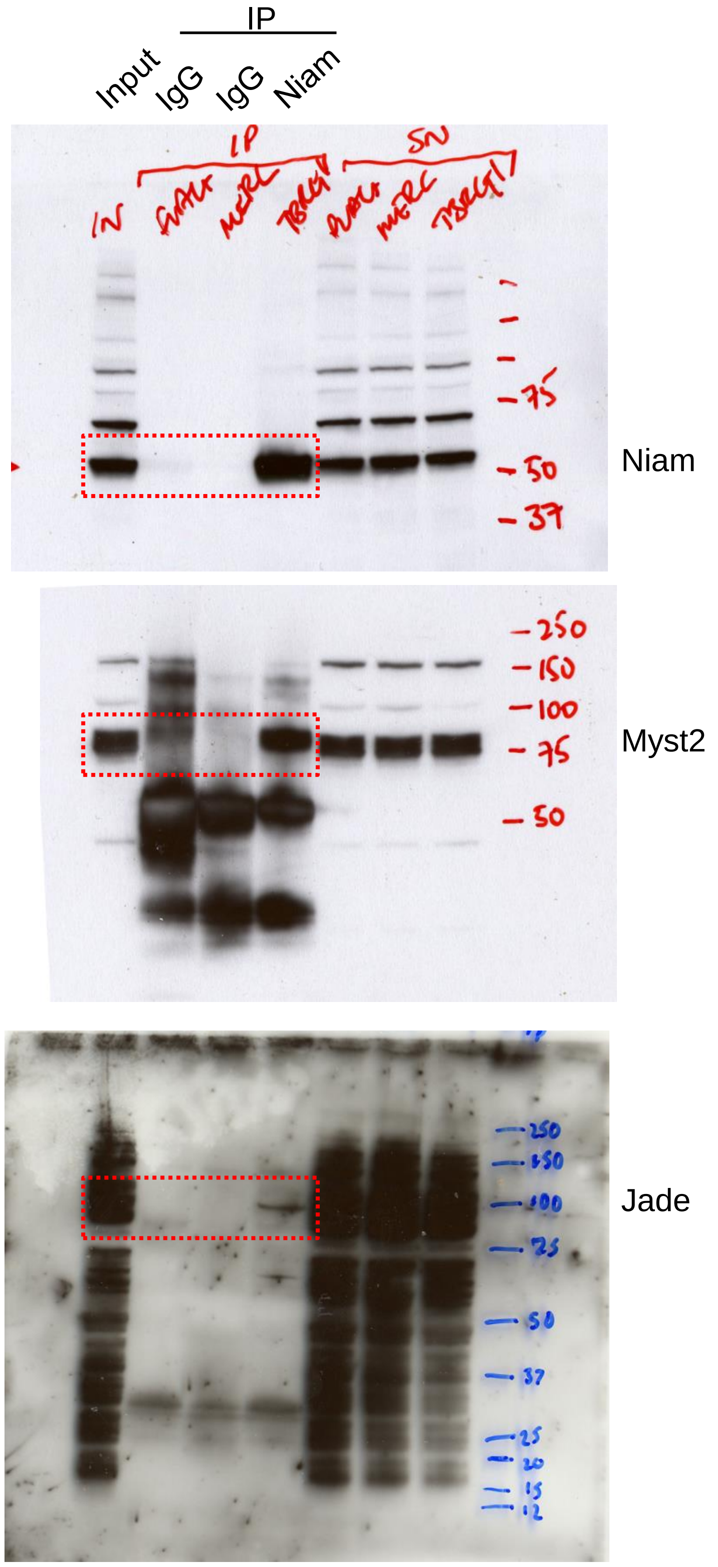

Supplementary Figure S4. Full-length images of western blots presented in Fig. 3a. The cropped images shown in Fig. 3a are marked here with a red dotted line.

Supplementary Figure S5

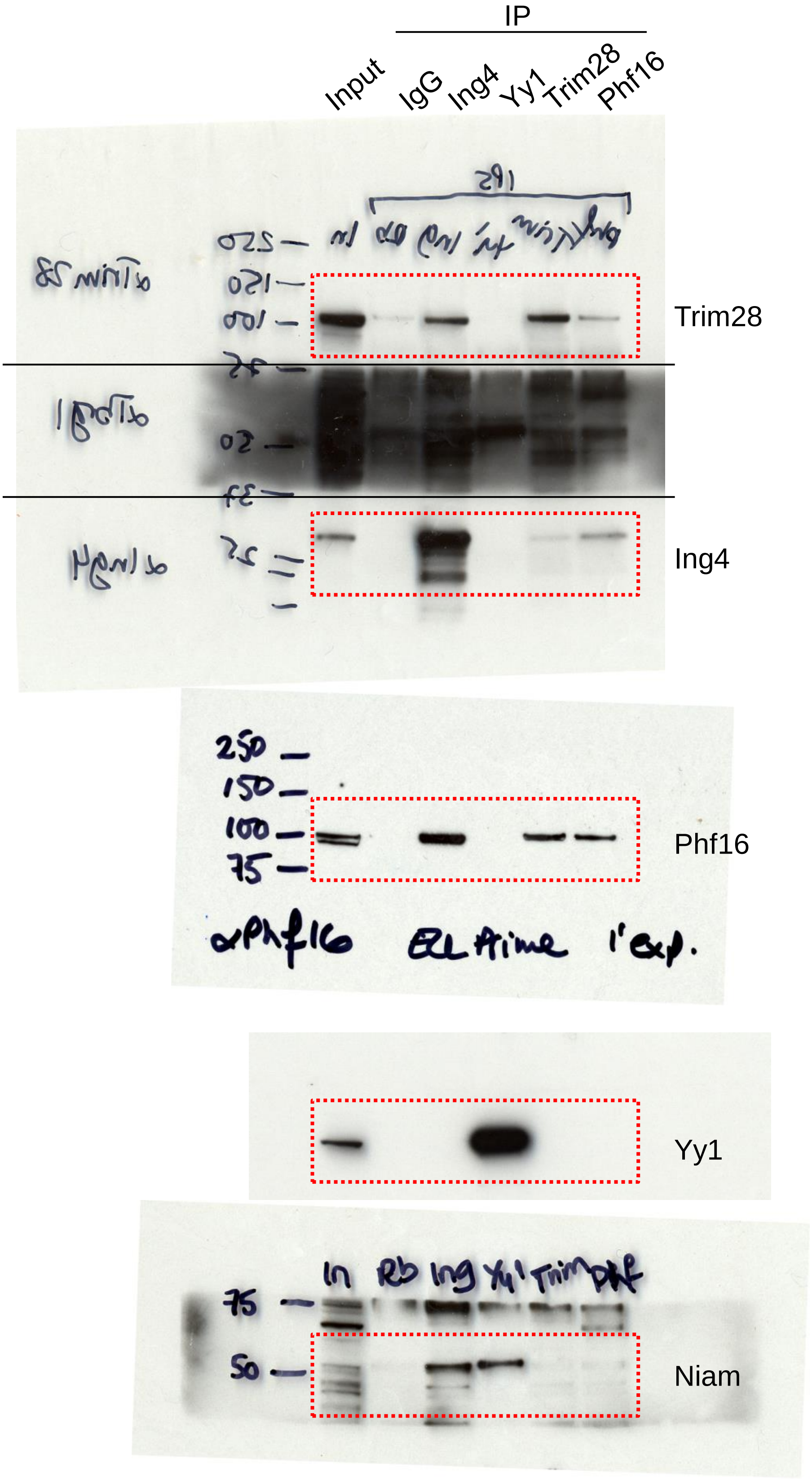

Supplementary Figure S5. Full-length images of western blots presented in Fig. 4b. The cropped images shown in Fig. 4b are marked here with a red dotted line.

Supplementary Table S1. Proteins identified from Myst2 TAP-MS experiments. Numbers of significant peptide sequences matched are shown for each TAP experiment.

**Accession**	**Description**	**Gene name**	**Exp I**	**Exp II**	**Exp III**
E9PZ26	Protein Brd1	Brd1	9	5	8
B2KF05	Bromodomain and PHD finger containing 3	Brpf3	32	25	33
Q60737	Casein kinase II subunit alpha	Csnk2a1	15	8	11
O54833	Casein kinase II subunit alpha’	Csnk2a2	2	3	4
G3UWL3	Casein kinase II subunit beta	Csnk2b	1	1	1
P97351	40S ribosomal protein S3a	Rps3a	1	8	2
P68433	Histone H3.1	Hist1h3a	2	2	2
P62806	Histone H4	Hist1h4a	4	3	3
Q8C0D7-2	Isoform 2 of inhibitor of growth protein 4	Ing4	5	4	8
Q9D8Y8-2	Isoform 2 of inhibitor of growth protein 5	Ing5	7	6	7
Q5SVQ0-3	Isoform 3 of Histone acetyltransferase KAT7	Myst2	22	26	44
Q9ERG0	LIM domain and actin-binding protein 1	Lima1	14	7	16
Q2VPQ9-2	Isoform 2 of Chromatin modification-related protein MEAF6	Meaf6	8	8	7
Q6ZQF7	Protein Jade-2	Phf15	10	11	12
B1AV39	PHD finger protein 16	Phf16	15	15	25

Q6ZPI0	Protein Jade-1	Phf17	28	26	28
Q9CR57	60S ribosomal protein L14	Rpl14	1	2	1
P62830	60S ribosomal protein L23	Rpl23	1	2	1
Q9JJI8	60S ribosomal protein L38	Rpl38	1	1	1
P60867	40S ribosomal protein S20	Rps20	3	5	1
Q3UB74	Transforming growth factor beta regulator 1	Niam	4	5	7
P05213	Tubulin alpha-1B chain	Tuba1b	4	1	1

Supplementary Table S2. Proteins identified from Niam IP-MS experiments. Numbers of significant peptide sequences matched are shown.

Accession	Description	Gene name	Exp I	Exp II	Exp III
E9Q607	Actin, cytoplasmic 2, N-terminally processed	Actg1	4	16	22
Q9Z2N8	Actin-like protein 6A	Actl6a	8	11	14
A2ACC9	ARP5 actin-related protein 5 homolog (Yeast)	Actr5	5	14	22
Q8R2S9	Actin-related protein 8	Actr8	6	17	29
A2BDX0	Activity-dependent neuroprotective protein	Adnp	4	12	15
P56480	ATP synthase subunit beta, mitochondrial	Atp5b	3	4	1
Q8K2J4	Coiled-coil domain-containing protein 14	Ccdc14	4	9	4
Q60737	Casein kinase II subunit alpha	Csnk2a1	2	4	4
P70698	CTP synthase 1	Ctps1	1	2	2
Q9JIK5	Nucleolar RNA helicase 2	Ddx21	21	4	9
F8WJA0	ATP-dependent RNA helicase DDX24	Ddx24	4	1	1
Q921N6	Probable ATP-dependent RNA helicase DDX27	Ddx27	4	1	1
Q61656	Probable ATP-dependent RNA helicase DDX5	Ddx5	2	5	10

Q6NZL1	DEAH (Asp-Glu-Ala-His) box polypeptide 37	Dhx37	2	2	6
E9QNN1	ATP-dependent RNA helicase A	Dhx9	17	6	20
Q9JHU4	Cytoplasmic dynein 1 heavy chain 1	Dync1h1	1	1	2
P70372	ELAV-like protein 1	Elavl1	3	1	1
Q8CIB9	N-acetyltransferase ESCO2	Esco2	1	1	1
Q5SUS9	Ewing sarcoma breakpoint region 1	Ewsr1	2	2	2
P35550	rRNA 2~-O-methyltransferase fibrillarin	Fbl	6	2	3
Q61584	Fragile X mental retardation syndrome-related protein 1	Fxr1	7	1	16
Q8CI11	Guanine nucleotide-binding protein-like 3	Gnl3	8	1	17
P15864	Histone H1.2	Hist1h1c	11	3	5
P43277	Histone H1.3	Hist1h1d	12	3	6
P20029	78 kDa glucose-regulated protein	Hspa5	8	13	24
P63017	Heat shock cognate 71 kDa protein	Hspa8	15	22	38
O88477	Insulin-like growth factor 2 mRNA-binding protein 1	Igf2bp1	12	4	19
Q8C0D7	Inhibitor of growth protein 4	Ing4	1	1	1
Q6ZPV2	DNA helicase INO80	Ino80	11	29	40
Q99PT3	INO80 complex subunit B	Ino80b	3	5	6

Q8BHA0	INO80 complex subunit C	Ino80c	6	8	6
E9Q9Q0	INO80 complex subunit D	Ino80d	1	8	4
D3Z3H0	Protein Ino80e	Ino80e	3	6	6
Q8C5L6	Inositol polyphosphate 5-phosphatase K	Inpp5k	1	1	1
Q5SVQ0	Histone acetyltransferase KAT7	Kat7	2	12	17
Q80WE4	Kinesin-like protein KIF20B	Kif20b	12	99	51
P52293	Importin subunit alpha-2	Kpna2	4	4	6
G3UYN0	LINE-1 type transposase domain-containing protein 1	L1td1	7	4	1
Q99L90	Microspherule protein 1	Mcrs1	1	9	10
E9PW39	Putative helicase MOV-10	Mov10	4	1	2
Q7TPV4	Myb-binding protein 1A	Mybbp1a	7	5	23
P70670	Nascent polypeptide-associated complex subunit alpha	Naca	4	5	3
P09405	Nucleolin	Ncl	8	3	5
Q6PIJ4	Nuclear factor related to kappa-B-binding protein	Nfrkb	13	41	63
E9Q5C9	Protein Nolc1	Nolc1	7	18	10
Q9D6Z1	Nucleolar protein 56	Nop56	12	5	23
Q6DFW4	Nucleolar protein 58	Nop58	5	2	12

Q61937	Nucleophosmin	Npm1	1	12	23
Q9CPP0	Nucleoplasmin-3	Npm3	1	3	4
E9QAE4	Histone-lysine N-methyltransferase, H3 lysine-36 and H4 lysine-20-specific	Nsd1	2	1	3
Q9CZ44-3	Isoform 3 of NSFL1 cofactor p47	Nsfl1c	6	22	31
E9Q7G0	Protein Numa1	Numa1	12	9	2
Q9R0L6-2	Isoform 2 of Pericentriolar material 1 protein	Pcm1	3	13	11
Q6NS46	Protein RRP5 homolog	Pdcd11	13	1	1
E9Q3F7	Retrotransposon-derived protein PEG10	Peg10	1	2	1
B1AV39	PHD finger protein 16	Phf16	5	14	5
P08775	DNA-directed RNA polymerase II subunit RPB1	Polr2a	12	23	5
Q8CFI7	DNA-directed RNA polymerase II subunit RPB2	Polr2b	4	11	10
Q99KP6-2	Isoform 2 of Pre-mRNA-processing factor 19	Prpf19	1	5	12
Q9DAW6	U4/U6 small nuclear ribonucleoprotein Prp4	Prpf4	1	4	10
P61290	Proteasome activator complex subunit 3	Psme3	1	4	3
Q9DAE2	Protein Rbmxl2	Rbmxl2	4	1	6
Q9CXW4	60S ribosomal protein L11	Rpl11	3	4	6

P35979	60S ribosomal protein L12	Rpl12	10	2	6
Q9CPR4	60S ribosomal protein L17	Rpl17	5	1	6
P62717	60S ribosomal protein L18a	Rpl18a	8	1	2
P84099	60S ribosomal protein L19	Rpl19	2	1	1
P62830	60S ribosomal protein L23	Rpl23	3	3	7
P62751	60S ribosomal protein L23a	Rpl23a	3	3	6
P27659	60S ribosomal protein L3	Rpl3	6	2	11
Q9D8E6	60S ribosomal protein L4	Rpl4	12	1	9
P47911	60S ribosomal protein L6	Rpl6	10	2	3
F6TJZ4	Uncharacterized protein (Fragment)	Rpl7a	3	1	4
P51410	60S ribosomal protein L9	Rpl9	1	1	3
Q8VDS4	Regulation of nuclear pre-mRNA domain-containing protein 1A	Rprd1a	8	6	5
Q9CSU0	Regulation of nuclear pre-mRNA domain-containing protein 1B	Rprd1b	6	10	8
P62843	40S ribosomal protein S15	Rps15	1	1	4
P62983	Ubiquitin-40S ribosomal protein S27a	Rps27a	4	1	4
P97351	40S ribosomal protein S3a	Rps3a	8	4	14
P62702	40S ribosomal protein S4, X isoform	Rps4x	4	3	3
P62242	40S ribosomal protein S8	Rps8	11	3	1

P14206	40S ribosomal protein SA	Rpsa	1	2	1
Q91YK2	Ribosomal RNA processing protein 1 homolog B	Rrp1b	5	1	10
P60122	RuvB-like 1	Ruvbl1	15	27	7
Q9WTM5	RuvB-like 2	Ruvbl2	24	40	12
Q8CH25	SAFB-like transcription modulator	Sltm	1	4	12
Q8BTI8	Serine/arginine repetitive matrix protein 2	Srrm2	7	9	12
P32067	Lupus La protein homolog	Ssb	1	1	3
O88935	Synapsin-1	Syn1	1	4	6
Q3UB74	Transforming growth factor beta regulator 1	Niam	6	11	9
O08784	Treacle protein	Tcof1	1	15	3
E9Q5S5	TCF3 fusion partner homolog	Tfpt	1	3	3
Q01320	DNA topoisomerase 2-alpha	Top2a	16	1	5
Q62318	Transcription intermediary factor 1-beta	Trim28	8	8	9
P99024	Tubulin beta-5 chain	Tubb5	22	9	11
Q91VX2	Ubiquitin-associated protein 2	Ubap2	16	21	26
Q9WUP7	Ubiquitin carboxyl-terminal hydrolase isozyme L5	Uchl5	6	13	13
Q640M1	U3 small nucleolar RNA-associated protein 14 homolog A	Utp14a	9	18	15

Q01853	Transitional endoplasmic reticulum ATPase	Vcp	16	53	55
P61965	WD repeat-containing protein 5	Wdr5	1	2	2
Q00899	Transcriptional repressor protein YY1	Yy1	1	7	15
Q80VJ6	Zinc finger and SCAN domain containing protein 4C	Zscan4c	3	5	6
E9Q5M0	Protein Zscan4e	Zscan4e	2	3	5

Supplementary Table S3. Enriched GO terms associated with Niam interactome. (See Excel file)

Supplementary Table S4. Loss-of-function phenotypes associated to Myst2 and Niam preys. Annotations were retrieved from the MGI database. Embryonic phenotypes are marked. (See Excel file)

Supplementary Table S5. Orthologues of Myst2-Niam network nodes with known cancer-causing somatic mutations or whose mutation results in genetic human disease. Annotations were retrieved from the COSMIC and OMIM databases.

Gene Name	OMIM description
ADNP	Helsmoortel-Van Der Aa Syndrome
CSNK2A1	Okur-Chung Neurodevelopmental Syndrome
DYNC1H1	Charcot-Marie-Tooth Disease Axonal Type 2o
DYNC1H1	Mental Retardation Autosomal Dominant 13
DYNC1H1	Spinal Muscular Atrophy Lower Extremity-Predominant 1 Autosomal Dominant
ESCO2	Roberts Syndrome

ESCO2	Sc Phocomelia Syndrome
EWSR1	Ewing Sarcoma
NOP56	Spinocerebellar Ataxia 36
NSD1	Beckwith-Wiedemann Syndrome
NSD1	Sotos Syndrome 1
NUMA1	Acute Promyelocytic Leukemia
PCM1	Thyroid Cancer Nonmedullary 1
PRPF4	Retinitis Pigmentosa 70
RPL11	Diamond-Blackfan Anemia 7
RPSA	Asplenia Isolated Congenital
SYN1	Epilepsy X-Linked With Variable Learning Disabilities And Behavior Disorders
TCOF1	Treacher Collins Syndrome 1
TUBB	Cortical Dysplasia Complex With Other Brain Malformations 6
TUBB	Skin Creases Congenital Symmetric Circumferential 1
VCP	Amyotrophic Lateral Sclerosis 14 With Or Without Frontotemporal Dementia
VCP	Charcot-Marie-Tooth Disease Axonal Type 2y
VCP	Inclusion Body Myopathy With Early-Onset Paget Disease With Or Without Frontotemporal Dementia 1
	**Tumour types**
DDX5	Prostate cancer
EWSR1	Ewing sarcoma; desmoplastic small round cell tumour ; ALL; clear cell sarcoma; sarcoma; myoepithelioma; mesothelioma
NACA	NHL
NPM1	NHL; APL; AML
NSD1	AML
NUMA1	APL
PCM1	papillary thyroid; CML; MPN
TFTP	pre-B ALL
